# Investigation of the Geochemical Preservation of *ca.* 3.0 Ga Permineralized and Encapsulated Microfossils by Nanoscale Secondary Ion Mass Spectrometry

**DOI:** 10.1089/ast.2016.1531

**Published:** 2017-12-01

**Authors:** Frédéric Delarue, François Robert, Kenichiro Sugitani, Romain Tartèse, Rémi Duhamel, Sylvie Derenne

**Affiliations:** ^1^ IMPMC Sorbonne Universités–MNHN, UPMC Univ Paris 06, UMR CNRS 7590, IRD UMR 206, Paris, France.; ^2^ Department of Environmental Engineering and Architecture, Graduate School of Environmental Studies, Nagoya University, Nagoya, Japan.; ^3^ Sorbonne Universités, UPMC Univ Paris 06, CNRS, UMR 7619 METIS, Paris, France.

## Abstract

Observations of Archean organic-walled microfossils suggest that their fossilization took place through both encapsulation and permineralization. In this study, we investigated microfossils from the *ca.* 3.0 Ga Farrel Quartzite (Pilbara, Western Australia) using transmitted light microscopy, scanning electron microscopy, Raman microspectrometry, and nanoscale secondary ion mass spectrometry (NanoSIMS) ion microprobe analyses. In contrast to previous studies, we demonstrated that permineralized microfossils were not characterized by the micrometric spatial relationships between Si and C-N as observed in thin sections. Permineralized microfossils are composed of carbonaceous globules that did not survive the acid treatment, whereas encapsulated microfossils were characterized due to their resistance to the acid maceration procedure. We also investigated the microscale relationship between the ^12^C^14^N^-^ and ^12^C_2_^-^ ion emission as a proxy of the N/C atomic ratio in both permineralized and encapsulated microfossils. After considering any potential matrix and microtopography effects, we demonstrate that the encapsulated microfossils exhibit the highest level of geochemical preservation. This finding shows that the chemical heterogeneity of the microfossils, observed at a spatial resolution of a few hundreds of micrometers, can be related to fossilization processes. Key Words: Carbonaceous matter—Farrel Quartzite—Fossilization—NanoSIMS—Nitrogen—Permineralization. Astrobiology 17, 1192–1202.

## 1. Introduction

Microfossil-like structures have been reported in numerous Archean rocks (*e.g.,* Walsh, 1992; Schopf, 1993; Javaux *et al.,* 2010; Sugitani *et al.,* 2010). As a result of thermal alteration, however, their morphological features and their geochemical composition had often been severely modified, making the univocal identification of microorganisms and associated metabolism difficult. Microorganisms are generally thought to be fossilized through permineralization resulting from the “early infiltration and permeation of tissues by mineral-charged water” (Schopf, 1975). Consequently, the organic remnants are progressively replaced by silica or carbonates during mineralization. However, the presence of organic-walled microfossils in some Archean rocks (Grey and Sugitani, 2009; Javaux *et al.* 2010; Sugitani *et al.,* 2015) points to the existence of other mechanisms of fossilization, such as encapsulation of microorganisms through, for example, nucleation of adjacent mineral crystals (Rainey and Jones, 2010). Through investigation of mineralization of microbial mats from Icelandic hot springs, Konhauser and Ferris (1996) proposed that encapsulation favors morphological and geochemical preservation of microorganisms. This is supported by experimental silicification of modern microorganisms, which shows that the negative effect on geochemical preservation caused by thermal alteration can be counterbalanced by encapsulation (Picard *et al.,* 2015). However, encapsulation has not yet been directly documented in Archean rocks despite its great potential for preservation of microorganisms.

Bulk N/C atomic ratio has been classically used as a proxy to characterize the preservation status of organic matter (Watanabe *et al.,* 1997; Beaumont and Robert, 1999). However, this bulk geochemical approach neglects potential heterogeneities in preservation among different organic remnants. Recent technological developments, notably in the field of secondary ion mass spectrometry, have allowed *in situ* elemental and isotopic investigations of putative microfossils at the micro- to nanoscale (Oehler *et al.,* 2009; House *et al.,* 2013). Notably, it has been shown that the ^12^C^14^N^-^/^12^C_2_^-^ molecular ionic ratio is strongly correlated with bulk N/C atomic ratio (Thomen *et al.,* 2014; Alleon *et al.,* 2015), opening up the possibility to evaluate the geochemical preservation of Archean microfossils at the micrometer scale. In pioneering studies, Oehler *et al.* (2009, 2010) used nanoscale secondary ion mass spectrometry (NanoSIMS) analyses to calculate *in situ* N/C atomic ratios ranging from *ca*. 0.0125 to 0.05 for Archean spheroid microfossils from 3.0 Ga cherts. However, the possible effects of microtopography on the ^12^C^14^N^-^/^12^C_2_^-^ molecular ratios determined for the microfossils studied by Oehler *et al.* (2009, 2010) have not been thoroughly evaluated, and it is known that microtopography may induce relatively large changes in ^12^C^14^N^-^/^12^C_2_^-^ ratios even though precise quantifications are still incomplete (*e.g.,* Thomen *et al.,* 2014; Alleon *et al.,* 2015). These potential analytical pitfalls have to be addressed to further evaluate the significance of the relatively high *in situ* N/C atomic values determined by Oehler *et al.* (2009, 2010) for Archean microfossils. In this respect, a recent study has highlighted that silicification promoted the exceptional geochemical preservation of organic microfossils in the 1.88 Ga Gunflint cherts that have N/C atomic ratios up to *ca*. 0.25–0.30 (Alleon *et al.,* 2016), which is commensurable with the N/C ratios of modern cyanobacteria and is, by far, higher than the N/C ratios determined by Oehler *et al.* (2009, 2010).

In this study, our purposes are (i) to provide a procedure to determine the preservation status of organic microfossils by studying the relationship between the emissions of the ^12^C_2_^-^ and ^12^C^14^N^-^ molecular ions in pure organic standards, kerogens, and microfossils from both thin sections and acid maceration residues and (ii) to discuss the effect of the process of fossilization, that is, permineralization versus encapsulation, on the geochemical preservation of microfossils from the 3.0 Ga Farrel Quartzite.

## 2. Material and Methods

### 2.1. Sample locality

A black chert sample was collected from the *ca*. 3.0 Ga Farrel Quartzite at the Mount Grant locality in the Goldsworthy greenstone belt, in the Pilbara Craton in Western Australia. The Farrel Quartzite is composed of a clastic formation up to 80 m thick containing fine-grained to very coarse-grained sandstone, including quartzite with minor conglomerate, mafic to ultramafic volcanoclastic layers, evaporite beds, and black chert layers (Sugitani *et al.,* 2007). This unit underwent greenschist facies metamorphism and was pervasively silicified. The *ca*. 30 cm thick microfossil-bearing black chert occurs in the uppermost part of the Farrel Quartzite and is closely associated with evaporite beds.

### 2.2. Analyses

#### 2.2.1. Sample preparations and microscopy

Transmission light microscopy (TLM) observations and NanoSIMS analyses were carried out on both rock thick sections (thickness of *ca*. 50 μm) and isolated kerogen, whereas scanning electron microscopy (SEM) observations were only performed on the kerogen. Kerogen isolation was performed on about 200 g of rock through successive demineralization using HF-HCl (Derenne *et al.,* 2008). Then, a few milligrams of kerogen was deposited on a microscope glass slide for TLM, SEM, and NanoSIMS investigations. Carbonaceous microfossils were first observed with TLM in order to define targets of interest. Then glass slides were directly gold coated (20 nm thick) for SEM energy dispersive X-ray spectroscopy analysis and imaging using a TESCAN VEGA II at the French National Museum of Natural History (MNHN) with an accelerating voltage of 15 kV.

#### 2.2.2. Raman microspectrometry

Raman spectra were obtained with a Renishaw InVIA microspectrometer, equipped with a 532 nm argon laser. The laser was focused on the sample by using a DMLM Leica microscope with a 50 × objective. The spectrometer was first calibrated with a silicon standard before the analytical session. For each target, we determined the Raman shift intensity in the spectral window from 1000 to 1900 cm^−1^ including the first-order disorder carbon (D) and graphite (G) bands. A laser power below 1 mW was used to prevent any thermal alteration during the spectra acquisition. Finally, spectra acquisition was achieved after three successive iterations using a time exposure of 40 s.

#### 2.2.3. Nanoscale secondary ion mass spectrometry

**FIG. 1.**
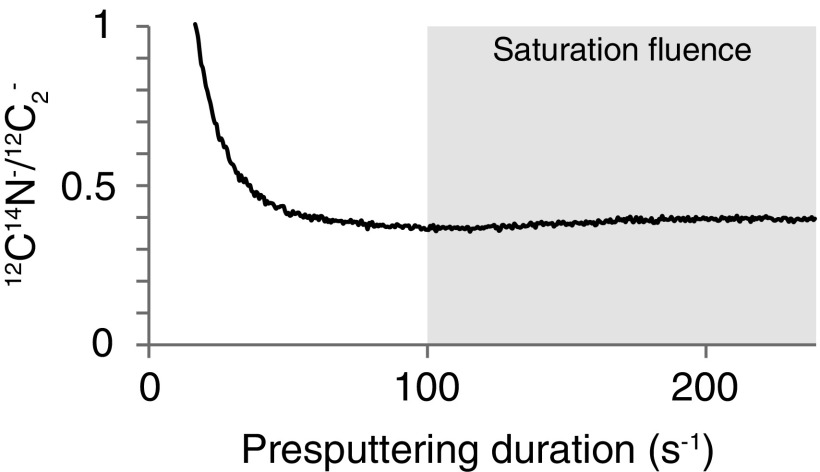
^12^C^14^N^-^/^12^C_2_^-^ ratio recorded as a function of the presputtering duration. The gray area indicates the time window in which the saturation fluence was achieved on the resin standard. Saturation fluence was systematically controlled for each studied microfossil.

Standards and microfossils were analyzed with the CAMECA NanoSIMS 50 at the MNHN. Before measurements, presputtering is required (i) to avoid surficial contamination and (ii) to achieve the saturation fluence of implemented cesium (Cs^+^) in order to obtain constant secondary ion count rates and then a constant ^12^C^14^N^-^/^12^C_2_^-^ ionic ratio (Fig. 1). Hence, Cs^+^ was implanted by using a 200 pA primary current (300 μm aperture diaphragm) on 50 × 50 to 75 × 75 μm^2^ areas, depending of the size of each target. Analyses were then carried out by using a 5 pA primary current (150 μm aperture diaphragm) on smaller areas to avoid presputtering edge artifacts. Secondary molecular ions and species of ^12^C_2_^-^, ^12^C^14^N^-^, and ^28^Si^-^ were collected simultaneously in electron multipliers. NanoSIMS raw data were corrected for a 44 ns dead time on each electron multiplier and were processed with the Limage software (developed by L. Nittler, Carnegie Institution, Washington, DC, USA). The external reproducibility was determined through multiple measurements of the emissions of the ^12^C^14^N^-^ and ^12^C_2_^-^ molecules on a coal standard used by Thomen *et al.* (2014). A second NanoSIMS session was dedicated to the analyses of a blank (polycarbonate filter), pure organic standards (resin and tryptophan), and a type III kerogen (land plant–derived carbonaceous matter). These pure standards and the type III kerogen correspond to the standards previously used in the work of Alleon *et al.* (2015). Hence, Cs^+^ was implanted by using a 400 pA primary current (150 μm aperture diaphragm) on 45 × 45 μm^2^ areas. Analyses were then carried out by using a 1 pA primary current (150 μm aperture diaphragm) on smaller areas to avoid presputtering edge artifacts.

#### 2.2.4. Statistics and errors

Correlations between the ^12^C_2_^-^ and ^12^C^14^N^-^ and ^28^Si^-^ ion emissions were tested with Spearman's rank correlation. A *p* value inferior to 0.05 is indicative of a significant correlation. In the presence of a significant spatial relationship between the emissions of ions, linear regressions were performed to calculate the value of the slope and its associated standard error (1σ_reg_) following 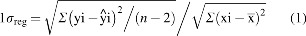


where yi is the emission of the ^12^C^14^N^-^ ion measured by NanoSIMS, ŷi is the emission of the ^12^C^14^N^-^ ion determined by linear regression, xi is the emission of the ^12^C_2_^-^ ion measured by NanoSIMS, ${ \rm{ \overline x}}$ is the average value of the emissions of the ^12^C_2_^-^ ion, and where *n* is the number of regions of interest (ROIs).

The external reproducibility was determined by determining the slope of the regression line between the ^12^C_2_^-^ and ^12^C^14^N^-^ ion emissions of a coal standard (*n* = 7). Then, the standard error of the mean slope *α* (1σ_rep_) was calculated.

Finally, the total error (1σ_tot_) was determined as follows:

\begin{align*}
1{ \sigma _{{ \rm{tot}}}} = \sqrt {1 \sigma _{{ \rm{reg}}}^2 + 1 \sigma _{{ \rm{rep}}}^2} \tag{2}
\end{align*}

## 3. Results

### 3.1. Carbonaceous microfossils in thin section and kerogen

**FIG. 2.**
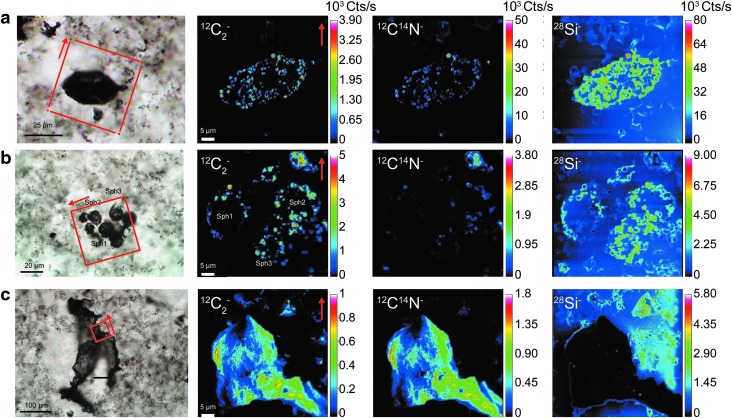
Transmitted light photomicrographs and NanoSIMS ion images (^12^C_2_^-^, ^12^C^14^N^-^, and ^28^Si^-^) of (**a**) a lenticular-like microfossil, (**b**) a spheroid cluster, and (**c**) a film-like microfossil. The microfossils were all observed on thin section. On each photomicrograph, the red dashed square outline indicates the area investigated by NanoSIMS.

A morphological diversity of microfossils was observed in thin section, with assemblages of lenticular (formerly described as spindle-like; *ca*. 20–40 μm; Sugitani *et al.,* 2007, 2009; Grey and Sugitani, 2009), film-like (>100 μm), and spheroidal (mainly <15 μm) microfossils occurring either as isolated specimens or as clusters (Fig. 2). In both spheroids and lenticular structures analyzed in thin sections, the ^12^C_2_^-^ and ^12^C^14^N^-^ ion emissions (Fig. 2a, 2b) are found within the siliceous matrix. In the film-like microstructure (Fig. 2c), the ^12^C_2_^-^ and ^12^C^14^N^-^ are emitted with almost no emission of ^28^Si^-^. This observation illustrates the fact that this microstructure was encapsulated by the siliceous matrix.

**FIG. 3.**
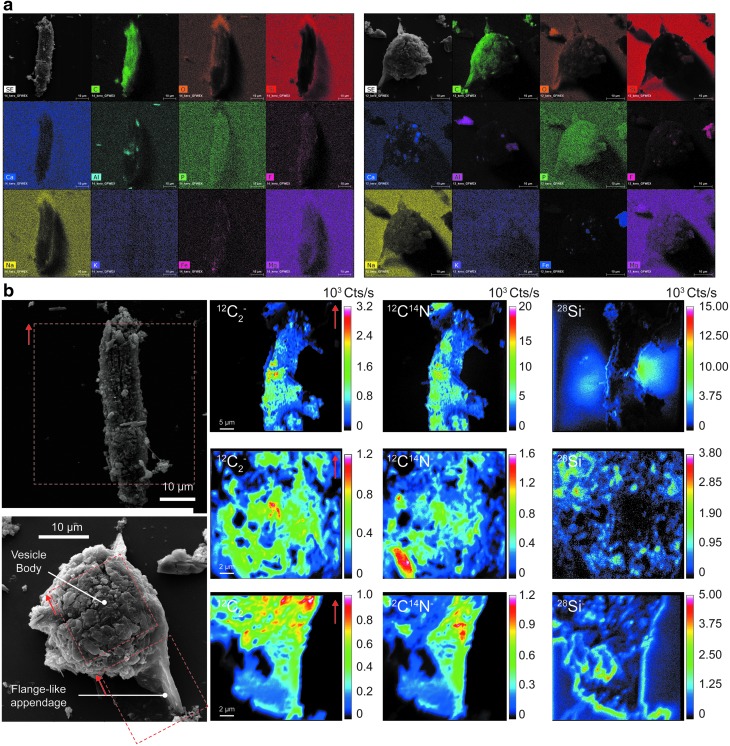
(**a**) Secondary electron and X-ray images of selected elements for a film-like and a well-preserved lenticular-like isolated microfossil. (**b**) NanoSIMS ion images (^12^C_2_^-^, ^12^C^14^N^-^, and ^28^Si^-^) determined on the filament-like microfossil and on the two distinct ultrastructures of the well-preserved lenticular-like microfossil, namely the vesicle body and the flange-like appendage.

**FIG. 4.**
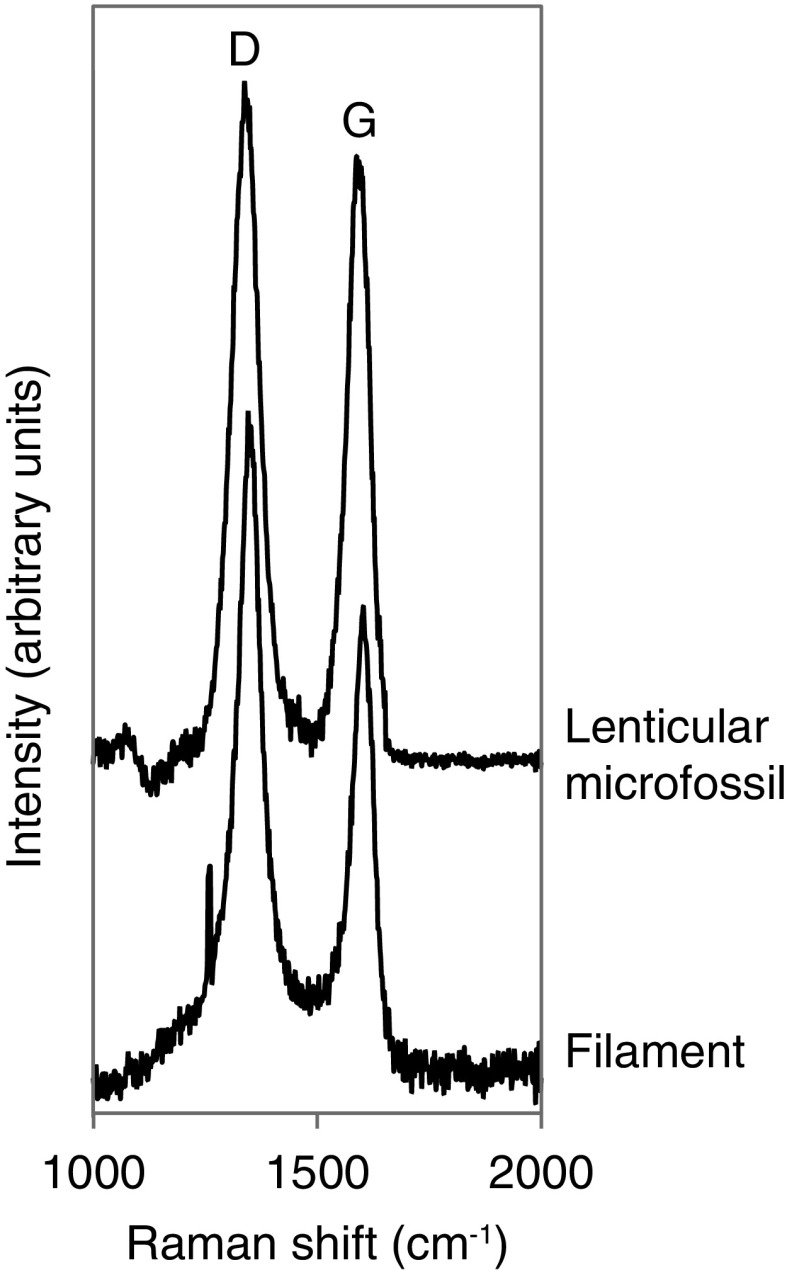
First-order Raman spectrum of the filament and the lenticular microfossil presented in Fig. 3.

Characterizing microfossils from thin sections with NanoSIMS implies that the analyzed targets occur at the very surface of the sample because the intensity of the primary beam cannot sputter more than a few atomic layers in depth. Therefore, the amount of microfossil targets in thin sections is limited. On the contrary, the kerogen fraction obtained by HF-HCl maceration of the fossil-bearing black cherts contains some microfossils morphologically equivalent to those in thin section (Grey and Sugitani, 2009). Although spheroids identified in the thin section were not found in the kerogen residue, lenticular and film-like microfossils were also observed in the kerogen fraction (Fig. 3). These lenticular and film-like microfossils are characterized by Raman line shape (Fig. 4),which is consistent with previous Raman spectra determined on microfossils from thin section (Sugitani *et al.,* 2007).

### 3.2. NanoSIMS quantitative investigation

The N/C atomic ratio has been classically used to assess the preservation status of ancient organic matter (Watanabe *et al.,* 1997; Beaumont and Robert, 1999). For NanoSIMS analysis, the ^12^C^14^N^-^/^12^C_2_^-^ ionic ratio has been regarded as a proxy of the N/C atomic ratio for silica-free kerogens or pure organic standards (Thomen *et al.,* 2014; Alleon *et al.,* 2015). However, in the case of microfossils, two additional analytical artifacts should be considered as follows: (1) matrix effects (chemical heterogeneity) linked to the occurrence of silicate minerals and (2) microtopographic effects yielding a difference in the emissivity of the ^12^C_2_^-^ and ^12^C^14^N^-^ ions. Owing to the imaging capability of the NanoSIMS, the ^12^C_2_^-^ and ^12^C^14^N^-^ variations have been recorded at a high spatial resolution. In the following, we demonstrate that the spatial variation between ^12^C_2_^-^ and ^12^C^14^N^-^ is linear and that its corresponding slope *α* is correlated with the N/C atomic ratio. However, this linear variation between ^12^C_2_^-^ and ^12^C^14^N^-^ shows a nonzero intercept *β*, possibly related to the sample surface microtopography. No relation was found between *β* and *α,* justifying in turn the use of *α* to record the relative variations of the N/C ratio. In addition, no measurable variation in the emissivity of the ^12^C_2_^-^ and ^12^C^14^N^-^ ions has been detected in the presence of silicate minerals, avoiding measurable matrix effects on *α*.

#### 3.2.1. The slope *α,* a record of the N/C atomic ratio

**FIG. 5.**
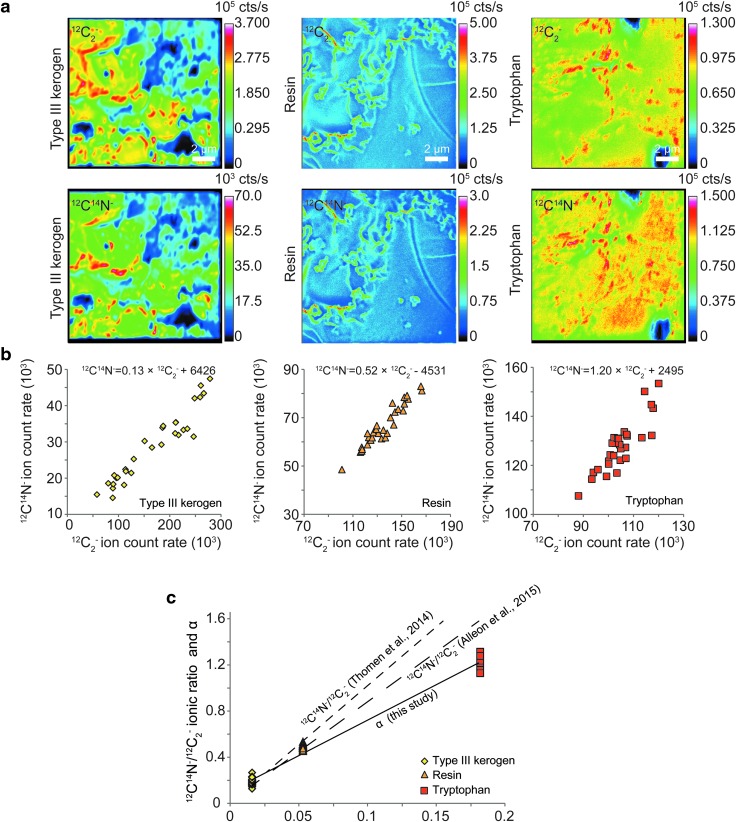
(**a**) NanoSIMS ion images (^12^C_2_^-^ and ^12^C^14^N^-^) of type III kerogen, resin, and tryptophan. (**b**) Relationship between the emissions of ^12^C_2_^-^ and ^12^C^14^N^-^ ions in each standard. Each point is related to a ROI (*n* = 30) manually drawn on the flattest part of the standards following the procedure described in Alleon *et al.* (2015; see Table 1 for further information about the number and size of ROIs). (**c**) Determination of the N/C atomic ratio by the use of the ^12^C^14^N^-^/^12^C_2_^-^ ionic ratio (Thomen *et al.,* 2014; Alleon *et al.,* 2015) and the slope *α*. Note that coal and microfossils (session 1) were not studied in the same analytical session as the three standards (session 2) used to calibrate the use of the slope *α* as a proxy of the N/C atomic ratio. Accordingly, this prevents any calculation of *in situ* N/C atomic ratios of microfossils. Also, note the linear relationship in each case, which evidences the absence of matrix effect on the determination of both ^12^C^14^N^-^/^12^C_2_^-^ ionic ratio and the slope *α*.

Emissions of ^12^C_2_^-^ and ^12^C^14^N^-^ in resin, tryptophan, and a type III kerogen are systematically correlated (Fig. 5a, 5b; Table 1). Although both emissions converge toward 0 for lower ion counting rates, the linear regression calculated on the whole range of emissions yields a nonzero intercept *β*. This relationship is characterized by a slope *α*. In Fig. 5c, the slopes of pure kerogen and standards are reported versus bulk N/C atomic ratio. Note that for a N-free sample (polycarbonate filter) no relationship between the emissions of the ^12^C_2_^-^ and ^12^C^14^N^-^ ions is found (Table 1). Hence, a significantly linear relationship between the ^12^C_2_^-^ and ^12^C^14^N^-^ ion emissions is the preliminary condition to consider in order to define a slope *α* that can be used to record the N/C atomic ratio.

**Table 1.** Bulk N/C Atomic Ratio (Thomen *et al.,*^2014^; Alleon *et al.,*^2015^) of Studied Standards, Number and Size of Regions of Interest (ROIs) Used (i) to Test the Correlation between the Emissions of the ^12^C_2_^-^ and ^12^C^14^N^-^ Ions by Using Spearman's Rank Correlation and (ii) to Determine the Slope *α* on Standards

*Purpose*	*Sample*	*Bulk N/C atomic ratio*	*Number of ROIs*	*ROIs diameter (μm)*	*Spearman* p *value*	α* ± 1σ_reg_*
Blank	Polycarbonate filter	—	30	1.5	0.50	—
Calibration line	Type III kerogen	0.016	30	1.5	<0.0001	0.13 ± 0.01
	Resin	0.053	30	0.8	<0.0001	0.52 ± 0.05
	Tryptophan	0.182	30	1.5	<0.0001	1.20 ± 0.13
External reproducibility	Coal 1	0.0022	545	1.1	<0.0001	0.11 ± 0.004
	Coal 2	0.0022	460	1.1	<0.0001	0.10 ± 0.002
	Coal 3	0.0022	457	1.3	<0.0001	0.09 ± 0.002
	Coal 4	0.0022	478	1.3	<0.0001	0.09 ± 0.004
	Coal 5	0.0022	321	1.3	<0.0001	0.13 ± 0.003
	Coal 6	0.0022	365	1.3	<0.0001	0.09 ± 0.002
	Coal 7	0.0022	367	1.4	<0.0001	0.08 ± 0.004

To minimize the effect of microtopography, ROIs were selected in the flattest part of the standard as recommended by Alleon *et al.* (2015). In the specific case of the coal standard, our purpose was to measure the external reproducibility. Thus, a maximum of ROIs were selected to characterize the strict effect of analytical drift, avoiding then to take into account twice the effect of microtopography during the analyses of microfossils. Note that the polycarbonate filter was used as a nitrogen blank in which the ^12^C_2_^-^ and ^12^C^14^N^-^ ion emissions are not significantly correlated.

#### 3.2.2. Matrix effect

**FIG. 6.**
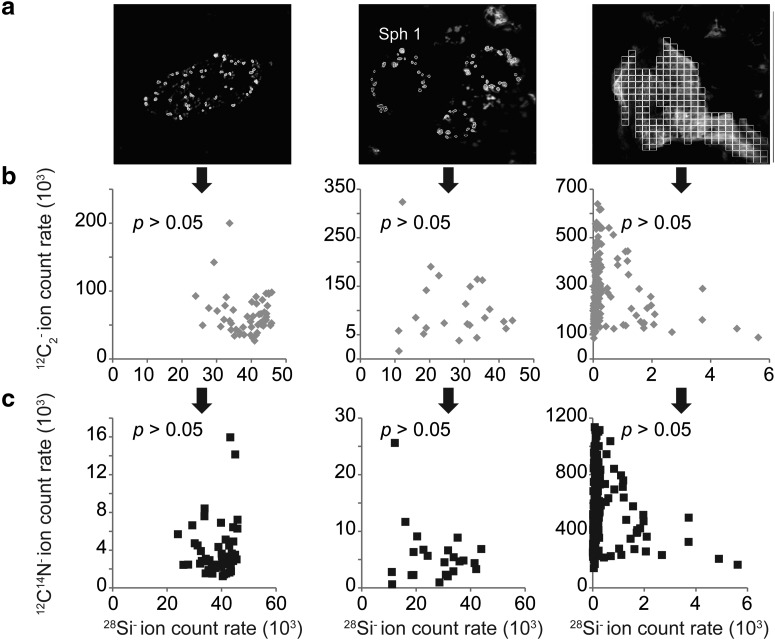
(**a**) NanoSIMS images (^12^C_2_^-^) of microfossils from thick section (presented in Fig. 2) displaying the ROIs used to study the potential relationship between (**b**) the ^28^Si^-^ and the ^12^C_2_^-^ ions and (**c**) the ^28^Si^-^ and the ^12^C^4^N^-^ ions. In order to constrain the spatial variability of the emissions of the ^12^C_2_^-^ and ^12^C^14^N^-^ ions, ROIs were manually drawn around carbon/silica in permineralized microfossils. For encapsulated microfossils, a grid was used (see Table 1 for further information on the number and the size of the ROIs).

Oehler *et al.* (2009) defined a matrix effect in their NanoSIMS measurements as the enhancement of the ^28^Si^-^ and ^16^O^-^ ion emissions when these ions are closely and spatially associated with carbon. Such a matrix effect may be linked to a higher conductibility of Si associated with carbonaceous globules compared to that of Si in the surrounding siliceous minerals. In microfossils from chert thick section (Fig. 6a), no relationship between ^28^Si^-^ on the one hand and ^12^C_2_^-^ (Fig. 6b) and ^12^C^14^N^-^ (Fig. 6c) ion emissions on the other hand has been found. Hence, *α* is not affected by the occurrence of Si.

#### 3.2.3. Microtopography

**FIG. 7.**
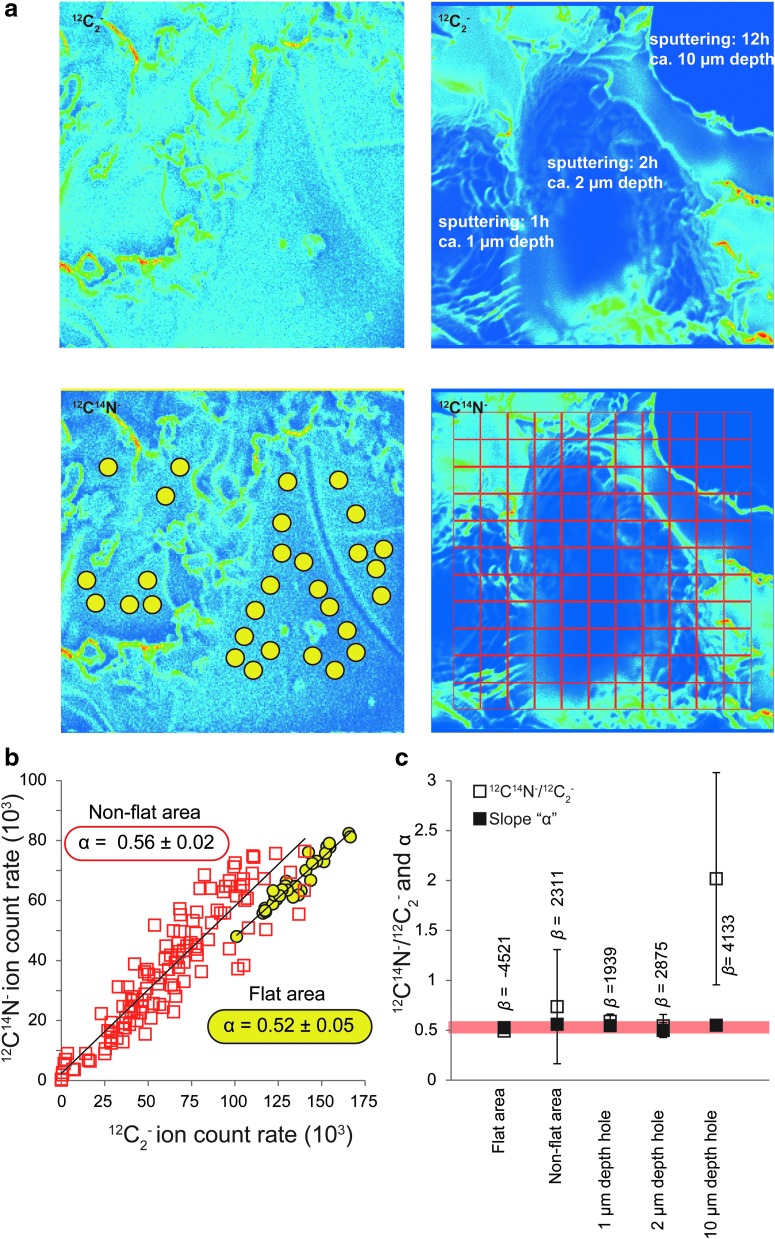
Investigation of the effect of microtopography on the determination of the slope *α* and its error on (**a**) ^12^C_2_^-^ and ^12^C^14^N^-^ ion flat (left images) and nonflat (right images) areas from the resin standards. In the nonflat area, photonic microtopography was created through extensive sputtering (1, 2, and 12 h), leading to the creation of micrometric-scale microtopography estimated through microscopy (*ca.* 1, 2, and 10 μm depth, respectively). The relationship between the emissions of ^12^C_2_^-^ and ^12^C^14^N^-^ ions was estimated on the above (i) flat area using 30 ROIs (yellow circles of 0.8 μm diameter) as suggested by Alleon *et al.* (2015) and (ii) nonflat area using 121 ROIs (red grid, each square has a width of 2.2 μm). (**b**) Relationships between the emissions of ^12^C_2_^-^ and ^12^C^14^N^-^ ions in the flat and the nonflat areas. (**c**) Comparison between the ^12^C^14^N^-^/^12^C_2_^-^ ionic ratio calculated as in Alleon *et al.* (2015) and the slope *α* (this study) values across microscale microtopography. The red area indicates the values of the slope *α* determined on the flat area. The intercept of the linear regression between the emissions of ^12^C_2_^-^ and ^12^C^14^N^-^ ions is noted *β*.

To investigate the effect of microtopography, we compared the ^12^C_2_^-^ and ^12^C^14^N^-^ ion emissions on two selected flat and nonflat (microtopographic features between 1 and 10 μm) areas from a chemically homogeneous resin standard (Fig. 7a). In Fig. 7, it can be seen that microtopography does not cause measurable shift in *α* (Fig. 7b; flat area: *α* = 0.52 ± 0.05; nonflat area: *α* = 0.56 ± 0.02).

Figure 7c shows that the value of the ^12^C^14^N^-^/^12^C_2_^-^ ratio is not affected by small micrometric scale microtopography up to 2 μm. Although a 10 μm of topography can enhance the ionic ^12^C^14^N^-^/^12^C_2_^-^ ratio by a factor of up to 4, the slope of the correlated variations between ^12^C^14^N^-^ and ^12^C_2_^-^ is only related to the N/C atomic ratio of the sample. Such a bias in the determination of the ^12^C^14^N^-^/^12^C_2_^-^ ratio is also related to the value of the nonzero intercept *β,* which seems to rise through enhanced microtopography (Fig. 7b, 7c). In contrast to the ^12^C^14^N^-^/^12^C_2_^-^ ratio, the slope *α* is constant in topographic domain covering 1–10 μm (Fig. 7c). Note that due to the size of the presently studied microfossils and the fact that microfossil edges were not considered, the microtopographic features cannot exceed a few micrometers.

Consequently, the matrix and microtopographic effects do not bias the use of the slope *α,* as a record of the N/C atomic ratio.

## 4. Discussion

Evidence for the permineralization of a part of the microfossils from the Farrel Quartzite was previously suggested by Oehler *et al.* (2009) owing to the co-emissions of the Si^-^, C_2_^-^, and CN^-^ ions in microfossils from thin section. Here, ^28^Si^-^ and ^12^C_2_^-^ ions on the one hand and ^28^Si^-^ and ^12^C^14^N^-^ ions on the other hand were not spatially associated at the pixel scale (Fig. 6). Such findings may echo results observed in the 3.4 Ga Strelley Pool Formation, in which Lepot *et al.* (2013) observed lenticular microfossils composed of carbonaceous globules that were interpreted as degradation by-products of Archean microorganisms. However, in the present study, no 3-D carbonaceous globules were observed in the isolated kerogen. Since they did not survive the acid treatment, they must not be considered encapsulated but rather permineralized.

In contrast to carbonaceous globules, carbonaceous microfossils were recovered in the acid maceration residue. Among these microfossils, one example of an exceptional morphological preservation of a lenticular microfossil is shown. Classically, in the literature, lenticular microfossils exhibit two kinds of flange-like appendages situated either in the equatorial plane or at the apical part of the vesicle body (Sugitani *et al.,* 2009; House *et al.,* 2013). Here, the flange-like appendage was situated at the apical part of the vesicle body. Lenticular, but also film-like, microfossils consist almost entirely of organic matter, suggesting that they are organic-walled microfossils removed from the silica matrix by the HF treatment. In turn, this result implies the preservation of some organic-walled microfossils by encapsulation rather than by permineralization. These organic-walled microfossils are characterized by equivalent Raman line shape (Fig. 4), corresponding to advanced carbonization/greenschist facies metamorphism in silicified cherts (Delarue *et al.,* 2016). Raman characteristics of the microfossils are then consistent with the thermal history of the Farrel Quartzite cherts (Sugitani *et al.,* 2007), revealing, in turn, their syngenicity.

**FIG. 8.**
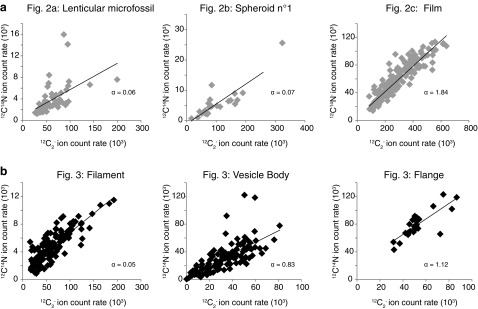
(**a**) Relationships between the emissions of ^12^C_2_^-^ and ^12^C^14^N^-^ ions in microfossils from thin section. (**b**) Relationship between the emissions of ^12^C_2_^-^ and ^12^C^14^N^-^ ions in encapsulated microfossils from the kerogen residue.

The slope *α* parameter for both permineralized and encapsulated microfossils was calculated as a proxy of the *in situ* N/C atomic ratio (Fig. 8; Table 2). The correlation between ^12^C_2_^-^ and ^12^C^14^N^-^ is statistically significant for 11 out of the 14 analyzed microfossils (Table 2), which will be considered in the following discussion.

**Table 2.** Number and Diameter of ROIs Used on Studied Microfossils

*Microfossil type*	*Thin section (TS) vs. kerogen (K)*	*Encapsulated (E) vs. permineralized (P)*	*Number of ROIs*	*ROI diameter (μm ± SD)*	*Spearman* p *value*	α* ± 1σ_tot_*
Lenticular	TS	P	25	0.63 ± 0.15	0.0013	0.19 ± 0.05
Lenticular^a,c^	TS	P	49	0.66 ± 0.12	<0.0001	0.06 ± 0.01
Lenticular	TS	P	42	0.78 ± 0.20	0.0002	0.07 ± 0.02
Spheroid	TS	P	52	0.66 ± 0.11	<0.0001	0.03 ± 0.01
Spheroid	TS	P	25	0.65 ± 0.40	0.14	—
Spheroid 1^a,c^	TS	P	23	0.94 ± 0.27	<0.0001	0.07 ± 0.01
Spheroid 2	TS	P	20	1.08 ± 0.52	0.01	0.11 ± 0.05
Spheroid 3	TS	P	13	0.70 ± 0.38	0.11	—
Film	TS	E	252	2.9	<0.0001	1.48 ± 0.13
Filament^b,c^	K	E	146	2.4	<0.0001	0.05 ± 0.00
Film	K	E	45	1.3	0.11	—
Film^a,c^	TS	E	195	2.2	<0.0001	1.84 ± 0.14
Lenticular (vesicle)^b,c^	K	E	121	1.2	<0.0001	0.83 ± 0.09
Lenticular (flange)^b,c^	K	E	28	0.7	<0.0001	1.12 ± 0.18

Spearman's rank correlation, slope *α* and associated error (1σ_tot_) determined through the linear relationship between the emissions of the ^12^C_2_^-^ and ^12^C^14^N^-^ ions in both permineralized and encapsulated microfossils from thin section and kerogen.

^a,b,c^ Microfossils presented in Figs. 2, 3, and 8 are indicated by the superscript a, b, and c, respectively.

In the permineralized lenticular and spheroid microfossils, *α* ranges from 0.03 to 0.19, whereas it ranges from 0.05 to 1.84 in the encapsulated lenticular and film-like ones (Table 2). First of all, these data suggest that there is an unexpected geochemical heterogeneity among the microfossils preserved in the 3.0 Ga Farrel Quartzite. Most of the encapsulated microfossils are characterized by a greater *α* compared to permineralized ones (Fig. 8; Table 2). This indicates that encapsulated microfossils present a higher geochemical preservation level than the permineralized ones and that the mode of fossilization may be a key controlling factor in the geochemical heterogeneity in the Farrel Quartzite carbonaceous matter. Finally, this difference in the extent of geochemical preservation between permineralized and encapsulated microfossils is consistent with observations made on modern microbial mats that show that microorganisms are better preserved through encapsulation (Konhauser and Ferris, 1996). Focusing future studies on Archean encapsulated microfossils may then provide the best geochemical evidence in the search for traces of early life.

## 5. Conclusion

In this study, we provide new lines of evidence that support the partial fossilization of carbonaceous microfossils through encapsulation in the *ca*. 3.0 Ga cherts from the Farrel Quartzite in the Pilbara Craton, Western Australia. Encapsulated microfossils were observed both in the thin section and in the kerogen fraction. Using the slope *α* parameter relating the ^12^C_2_^-^ and ^12^C^14^N^-^ NanoSIMS emissions as an index of geochemical preservation of the studied microfossils, we demonstrate that encapsulated microfossils present a higher level of geochemical preservation than permineralized ones. Thus, the mechanism of fossilization of microorganisms may be considered as a key controlling factor in preserving geochemical heterogeneity.

Overall, our results suggest that focusing *in situ* investigations on well-preserved encapsulated carbonaceous matter may provide the best chance to recover information on the earliest forms of terrestrial life that are likely to be lost in bulk investigations.

## Abbreviations Used

MNHN: National Museum of Natural History
NanoSIMS: nanoscale secondary ion mass spectrometry, nanoscale secondary ion mass spectrometer
ROIs: regions of interest
SEM: scanning electron microscopy
TLM: transmission light microscopy
